# Triterpenoid Dihydro-CDDO-Trifluoroethyl Amide Protects against Maladaptive Cardiac Remodeling and Dysfunction in Mice: A Critical Role of Nrf2

**DOI:** 10.1371/journal.pone.0044899

**Published:** 2012-09-17

**Authors:** Yifan Xing, Ting Niu, Wenjuan Wang, Jinqing Li, Siying Li, Joseph S. Janicki, Stacey Ruiz, Colin J. Meyer, Xing Li Wang, Dongqi Tang, Yuxia Zhao, Taixing Cui

**Affiliations:** 1 Shandong University Qilu Hospital Research Center for Cell Therapy, Key Laboratory of Cardiovascular Remodeling and Function Research, Jinan, China; 2 Department of Traditional Medicine, Qilu Hospital of Shandong University, Jinan, China; 3 Department of Cell Biology and Anatomy, University of South Carolina School of Medicine, Columbia, South Carolina, United States of America; 4 Department of Pharmacology, Reata Pharmaceuticals, Inc., Irving, Texas, United States of America; Cardiovascular Research Institute Maastricht, Maastricht University, The Netherlands

## Abstract

**Background and Aims:**

Nuclear factor E2-related factor 2 (Nrf2) appears to be an attractive therapeutic target for the treatment of cardiac disease. We investigated whether a synthetic triterpenoid derivative of dihydro-CDDO-trifluoroethylamide (dh404), a novel Nrf2 activator, protects against pathological cardiac responses to hemodynamic stress in mice.

**Methods:**

Cardiac maladaptive remodeling and dysfunction were established by transverse aortic constriction (TAC) in mice. Hypertrophic growth of rat neonatal cardiomyocytes was induced by angiotensin II (Ang II). Cell death of rat neonatal cardiomyocytes was induced with hydrogen peroxide (H_2_O_2_). Cellular proliferation of rat neonatal cardiac fibroblasts was induced by Ang II, norepinephrine (NE) and phenylephrine (PE). Protein expression was assessed by immunochemical staining and Western blots. Gene expression was determined by real time reverse transcription-polymerase chain reaction (Q-PCR).

**Results:**

TAC suppressed myocardial Nrf2 expression, increased myocardial 4-hydroxy-2-nonenal and 8-hydroxydeoxyguanosine levels, and induced cardiac hypertrophy, fibrosis and apoptosis, and overt heart failure and death in mice. Administration of dh404 inhibited the pathological cardiac remodeling and dysfunction, and reduced the mortality. Moreover, dhd404 elevated myocardial levels of Nrf2 and Nrf2 nuclear translocation with a dramatic suppression of the oxidative stress in the heart. Dh404 inhibited hypertrophic growth and death in primary culture of rat neonatal cardiomyocytes and suppressed proliferation in primary culture of rat neonatal cardiac fibroblasts. However, these effects of dh404 were blunted by knocking down of Nrf2.

**Conclusion:**

These findings demonstrate that dh404 prevents pathological cardiac remodeling and dysfunction by activating Nrf2, indicating a therapeutic potential of dh404 for cardiac disease.

## Introduction

Heart failure is the frequent consequence of sustained, abnormal neurohormonal and mechanical stress and remains a leading cause of death worldwide [1]. Pathological stress, such as hypertension, results in cardiac hypertrophy, myocardial apoptosis and fibrosis, altered microvascular structure, and chamber dilation, culminating in cardiac dysfunction and heart failure [2], [3]. Despite the recent advances in understanding the molecular and cellular processes that contribute to heart failure [4], [5], there remains the need for further development of effective therapy for the prevention of pathological cardiac remodeling and dysfunction.

It is firmly established that oxidative stress characterized by elevated levels of reactive oxygen species (ROS) plays a causative role in the pathogenesis of cardiovascular disease [6]–[8]. This being the case it is surprising that clinical trials have shown antioxidant vitamins to be ineffective or even harmful for the treatment of cardiovascular disease [9]–[12]. Although these studies have not examined cardiac disease or heart failure per se, it is possible that a therapeutic benefit of antioxidant therapy for the prevention of heart failure may not have been achievable using non-selective ROS scavenging. Accordingly, effective therapy may require the specific targeting of either the endogenous antioxidant defense system or the source of oxidative stress.

**Figure 1:**
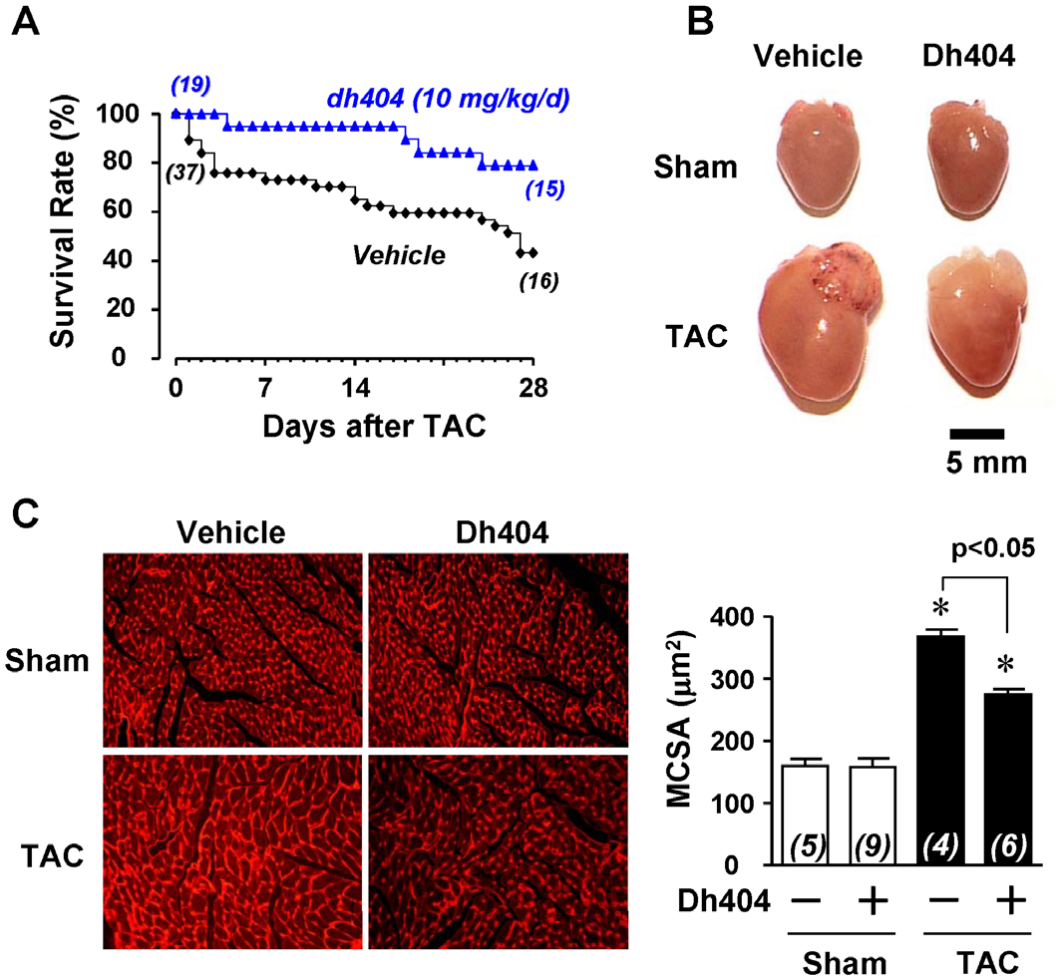
Effect of dh404 on survival rate and cardiac hypertrophy of mice after TAC. (A) Effect of dh404 (10 mg/kg/d) on TAC-induced death in C57BL/6J mice. (B) Representative pictures of hearts from sham and TAC operated mice treated with vehicle or dh404 (10 mg/kg/d). (C) Left panel - representative photomicrographs of left ventricular cross-sections depicting myocyte membranes stained with Texas Red-X conjugated wheat germ agglutinin (WGA). Right panel - quantitative left ventricular cardiac myocyte cross-sectional area (MCSA) from 2000 to 5000 myocytes per heart. The number of hearts (*n*) analyzed is indicated.

Nrf2 belongs to the Cap ‘n’ Collar (CNC) family of basic leucine zipper (bZip) transcription factors that include NF-E2, Nrf1–3 and Bach1–2 [13]. Nrf2 is a pleiotropic protein that binds to a *cis*-acting enhancer sequence known as the antioxidant response element (ARE) with a core nucleotide sequence of 5′-RTGACNNNGC-3′ to control the basal and inducible expression of a battery of antioxidant genes and other cytoprotective phase II detoxifying enzymes, such as heme oxygenase-1 (HO-1), superoxide dismutase (SOD), glutathione peroxidase (GPx), glutathione-S-transferases (GST), NAD(P)H:quinone oxidoreductase (NQO-1), NQO2, γ-glutamylcysteine synthase (γ-GCS), and glucuronosyltransferase. In addition to the wealth of evidence showing Nrf2 target genes such as HO-1 [14], [15], SOD [16], and GPx [17] to be cardioprotective, we have recently demonstrated it to be a critical endogenous inhibitor of maladaptive cardiac remodeling and dysfunction in the setting of pressure overload or sustained angiotensin II (Ang II) stimulation [18], [19]. We further demonstrated a down-regulation of Nrf2 protein expression in the failing heart of human diabetes [20]. While a recent report supporting a mediator role of Nrf2 in the antioxidant effects of hydrogen sulfide, an endogenous cardioprotective molecule [21], we and others have further revealed a pharmaceutical potential of targeting Nrf2 for the prevention of cardiomyocyte injury and cardiac dysfunction [22]–[26]. Collectively, Nrf2 appears to be a valuable therapeutic target for cardiac disease.

Thus, the purpose of this study was to determine whether the novel Nrf2 activator, which is a synthetic triterpenoid derivative of dihydro-CDDO-trifluoroethyl amide (dh404) [24], inhibits pressure overload-induced myocardial oxidative stress, maladaptive cardiac remodeling and heart failure in mice. Our results indicate that targeting Nrf2 by dh404 has significant potential to be a novel therapeutic approach for the prevention of adverse myocardial remodeling that leads to heart failure.

**Figure 2:**
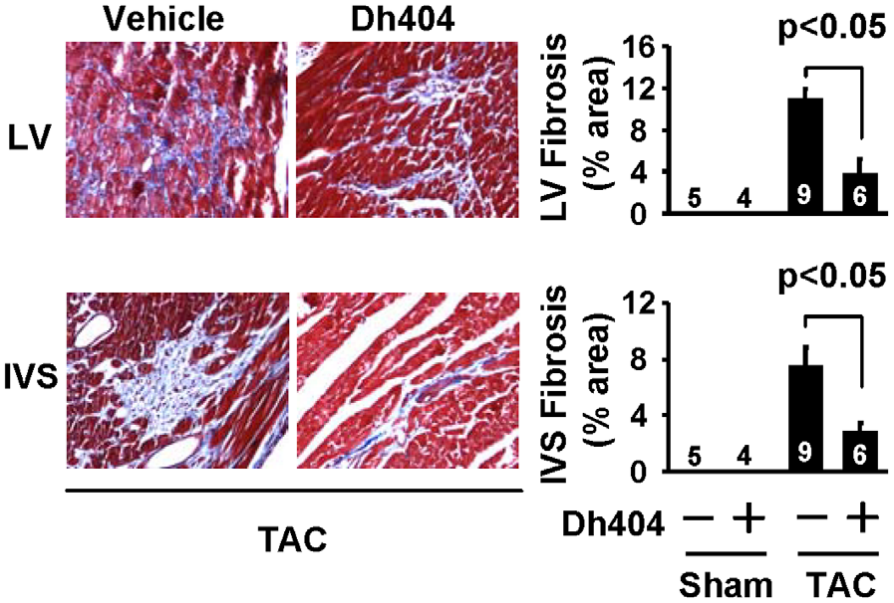
Effect of dh404 on myocardial fibrosis in mice after TAC. Myocardial fibrosis was assessed by staining of collagen with a Masson’s Trichrome Kit. In the left panels representative photomicrographs of a left ventricular (LV) and interventricular septum (IVS) sections from TAC hearts with and without dh404 treatment are presented. In the right panel the fibrotic areas of LV and IVS interstitial fibrosis as a percentage of total microscopic area per heart are presented. The numbers represent the number of sham and TAC hearts analyzed.

## Materials and Methods

### Ethics Statement

All procedures involving rat and mice were conducted in accordance with National Institutes of Health regulations concerning the use and care of experimental animals. All of the animal studies were approved by the University of South Carolina Animal Use Committee.

**Figure 3:**
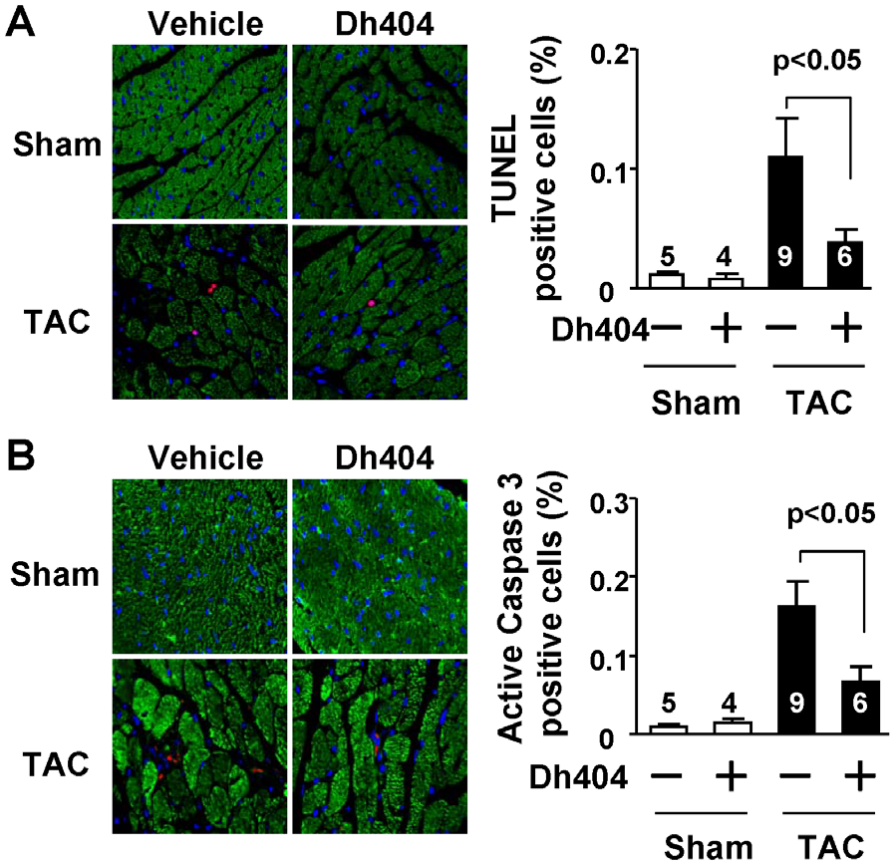
Effect of dh404 on myocardial apoptosis in mice after TAC. (A) In the left panel, the representative TUNEL staining of apoptotic cells in left ventricle are shown. TUNEL-positive, i.e., apoptotic nuclei in TAC hearts are stained red, nuclei blue and the myocardium is green (Alexa Fluor 488 Phalloidin to mark F-actin). Magnification, X 630. In the right panel, the apoptotic index results are given. TUNEL-positive cells were quantified as a percent of all nuclei in the section of LV. The number of hearts analyzed for each group is indicated. (B) In the left panel, the representative staining of cleaved caspase-3 positive cells in the left ventricle are shown. Cleaved caspase-3-positive, (i.e., cells with activated caspase-3) in TAC hearts are stained red and nuclei are blue. Cardiomyocytes were green utilizing an antibody of anti-cardiac myosin heavy chain. Magnification, X 630. In the right panel, the relative caspase-3 activity is summarized. Cleaved caspase-3-positive cells were quantified as a percent of all in a section of LV. The number of hearts analyzed for each group is indicated.

**Figure 4:**
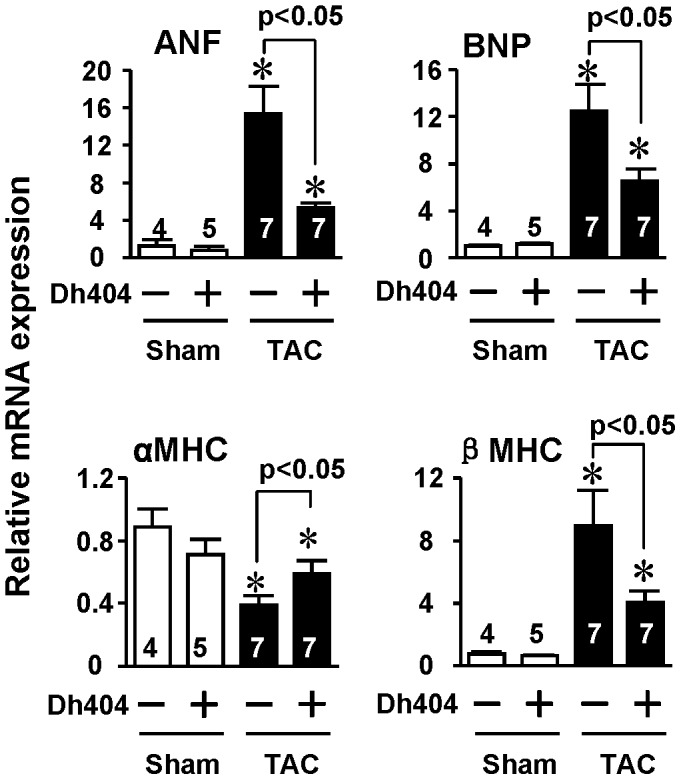
Effect of dh404 on myocardial fetal gene expression of mice after TAC. Hearts of mice were harvested, and left ventricles were dissected for RNA purification 4 weeks after the operation. Expression of ANF, BNP, αMHC, and βMHC was quantified by Q-PCR. *p<0.05 vs sham.

### Animals and Drug Treatment

Male C57BL/6J mice were purchased from JAX. Mice were housed under standard conditions in the Institution’s AAALAC approved animal facility. Dh404 was synthesized, purified, and characterized as previously described [24]. It was suspended in sesame oil (Sigma-Aldrich, S3547) and administrated by gavage at doses of 5 ∼ 20 mg/kg/d to C57BL/6J mice. The drug treatment was started one day prior to the pressure overload by transverse aortic arch constriction (TAC) and continued for an additional 4 wk.

**Table 1:** Echocardiography and pathology of mice treated with and without dh404 for 4 wks after TAC.

		Dh404 (mg/kg/d)			
		0	5	10	20
**Sham**	**Echocardiography** (n)	10	4	7	4
	IVSd (mm)	0.77±0.04	0.74±0.05	0.84±0.05	0.83±0.03
	LVIDd (mm)	4.06±0. 13	4.14±0.13	3.96±0.22	3.93±0.23
	LVIDs (mm)	4.06±0. 13	4.14±0.13	3.96±0.22	3.93±0.23
	LVPWd (mm)	0.69±0.03	0.64±0.02	0.68±0.02	0.65±0.05
	FS (%)	21.81±1.78	22.53±3.20	24.03±2.01	19.35±2.00
	**Pathology** (n)	8	4	6	3
	BW (g)	25.43±0.79	26.05±1.46	25.53±1.10	25.03±0.50
	HW/Tibia-L (g/cm)	7.52±0.29	7.61±0.53	7.40±0.34	7.02±0.17
	LW/Tibia-L (g/cm)	10.61±0.29	10.21±0.84	11.03±0.23	8.96±0.82
**TAC**	**Echocardiography** (n)	16	5	13	8
	IVSd (mm)	1.14±0.04^a^	0.91±0.06a,b	0.99±0.03a,b	0.94±0.05a,b
	LVIDd (mm)	5.11±0.14^a^	4.73±0.17a,b	4.73±0.11a,b	4.71±0.17a,b
	LVIDs (mm)	5.11±0.14^a^	4.73±0.17a,b	4.73±0.11a,b	4.71±0.17a,b
	LVPWd (mm)	1.08±0.04^a^	0.92±0.06a,b	0.90±0.03a,b	0.90±0.08a,b
	FS (%)	7.02±1.10^a^	7.88±1.40a,b	10.42±0.94a,b,c	14.77±1.97a,b,c,d
	**Pathology** (n)	16	5	13	8
	BW (g)	22.85±0.73^a^	23.56±0.80^a^	22.34±0.67^a^	20.65±0.97^a^
	HW/Tibia-L (g/cm)	15.54±0.48^a^	12.23±0.65a,b	11.68±0.42a,b	11.56±0.37a,b
	LW/Tibia-L (g/cm)	26.55±2.23^a^	15.79±4.30a,b	17.23±1.21a,b	21.17±2.76a,b

Two dimension guided M-mode echocardiography and pathology were performed 4 weeks after the initiation of TAC or sham surgery in C57BL/6J mice treated with vehicle or dh404 as indicated.

Abbreviations: IVSd, interventricular septum diastolic; LVIDd, left ventricular internal dimension diastolic; LVIDs, left ventricular internal septum diastolic; LVPWd, left ventricular posterior wall diastolic; FS, fractional shortening; BW, body weight; HW/Tibia L, heart weight/tibia length ratio; LW/Tibia-L, lung weight/tibia length ratio.

a p<0.05 vs sham (0);

b p<0.05 vs TAC (0);

c p<0.05 vs TAC + dh404 (5 mg/kg/d);

d p<0.05 vs TAC + dh404 (10 mg/kg/d).

### Transverse Aortic Arch Constriction (TAC) Model

TAC was induced in 8 to 10 wk old male mice and cardiac remodeling and dysfunction were measured as previously described [18]. Briefly at the experimental end-point, myocardial expression of fetal genes including atrial natriuretic factor (ANF), brain natriuretic peptide (BNP), alpha-myosin heavy chain (α-MHC), and beta-myosin heavy chain (β-MHC) was determined by quantitative real time reverse transcription-polymerase chain reaction (Q-PCR). Oxidative stress was assessed by 4-Hydroxy-2-Nonenal (4-HNE) and 8 hydroxydeoxyguanosine (8-OHdG) staining. Protein expression was determined by immunochemical staining and Western blot. Echocardiography was performed using the Vevo 770 High-Resolution Imaging System (VisualSonics Inc) with a 30-MHz transducer.

**Figure 5:**
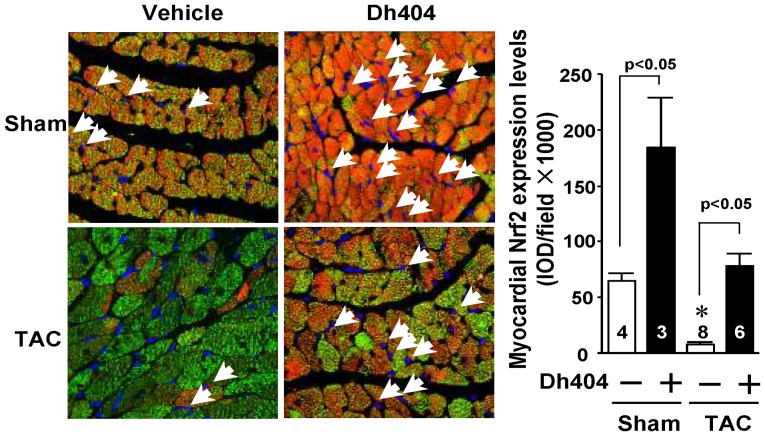
Effect of dh404 on myocardial Nrf2 expression of mice after TAC. C57BL/6J mice at age of 8 weeks were treated with vehicle or dh404 (10 mg/kg/d) and subjected to sham or TAC operations. Their hearts were harvested 4 weeks after the operation and analyzed for Nrf2 protein expression. Left panel - representative immunohistochemical staining of Nrf2 protein expression in left ventricles from TAC mice with or without dh404 treatment. Nrf2 nuclear translocation is indicated by arrows. Nrf2 staining was performed in 4 or more sections per heart. Nrf2 is stained red, nuclei blue and cardiomyocytes green utilizing an antibody of anti-cardiac myosin heavy chain. Right panel - a semi-quantitative analysis of Nrf2 protein levels in the heart. The number of hearts analyzed is indicated.

### Cell Cultures and Adenoviral Infection

Rat neonatal cardiac myocytes and fibroblasts were isolated and cultured as previously described [18]. The cells were infected with adenovirus of control and Nrf2 shRNAs as previously described [18]. Cardiomyocyte hypertrophic growth was assessed by [^3^H]leucine uptake. Cardiac fibroblast proliferation was determined by [^3^H]thymidine uptake. Cell death was measured using a Cytotoxicity Detection Kit (Roche Applied Science) as previously described [23].

**Figure 6:**
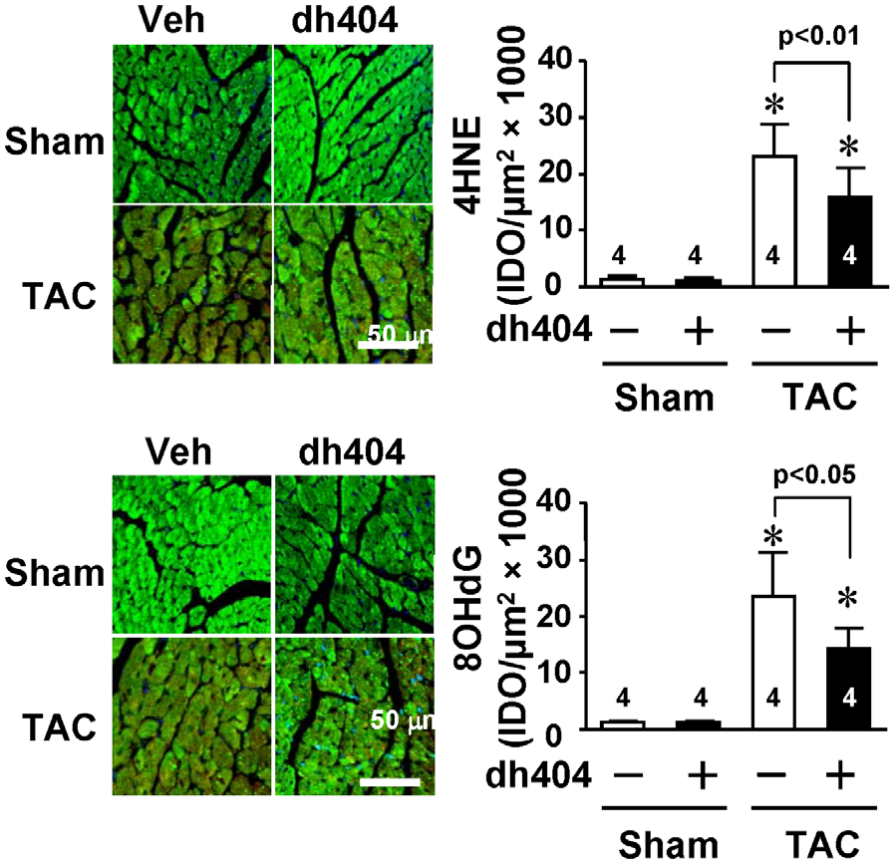
Effect of dh404 on myocardial oxidative stress of mice after TAC. Eight wk old C57BL/6J mice were grouped and treated as described in Figure 5. Left panel - representative confocal microscopic images of left ventricular 4-HNE staining and 8-OHdG staining. Areas positive for 4-HNE and 8-OHdG are shown in red, nuclei in blue were labelled with DAPI, and. cardiomyocytes in green were marked using anti-Tropomyosin I antibody against cardiac myocyte tropomyosin. Right panel - levels of 4-HNE and 8-OHdG were semi-quantified by measuring IOD of eight randomly chosen fields in each myocardial tissue section. The number of hearts (*n*) analyzed is indicated.

### Statistical Analysis

Data are shown as mean ± SD. Group comparisons were made using ANOVA followed by Bonferroni test for multiple comparisons. Differences were considered significant at p<0.05.

**Figure 7:**
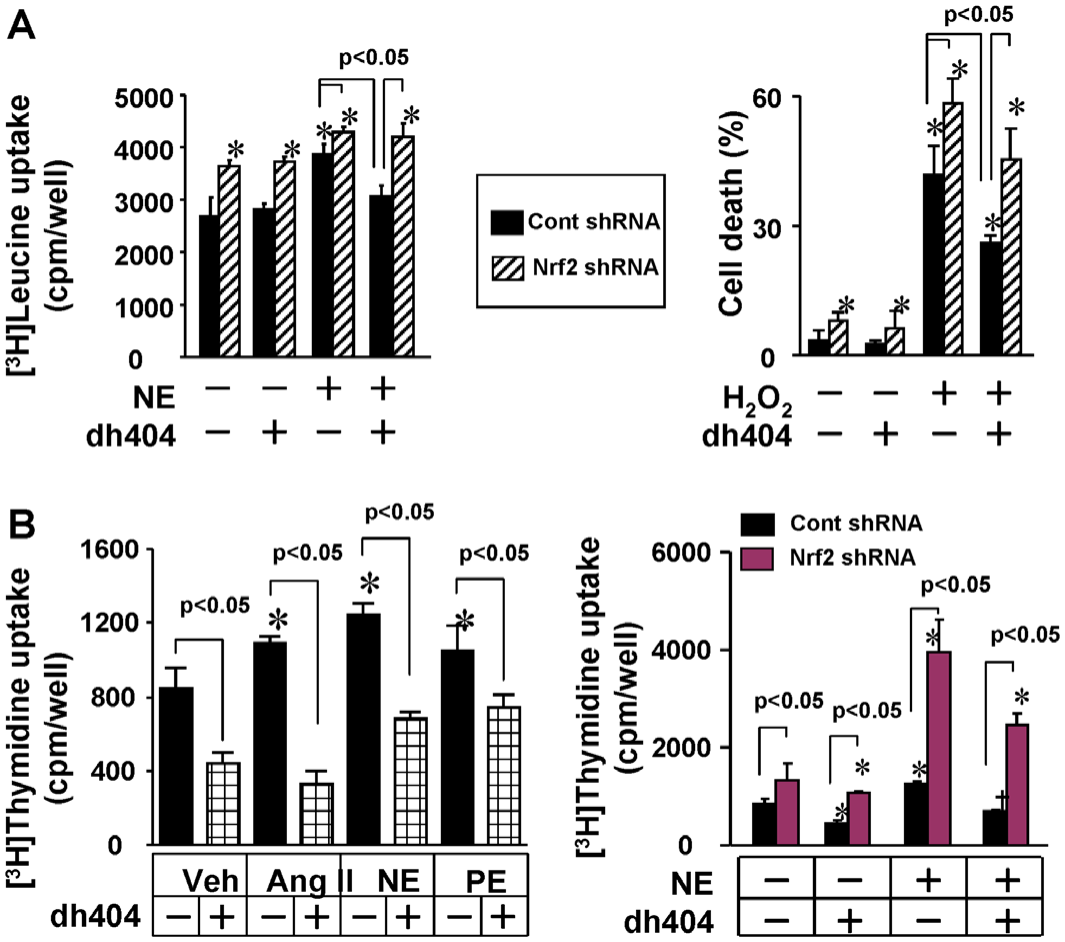
Role of Nrf2 in dh404-induced suppression of cardiomyocyte hypertrophy and death, and cardiac fibroblast proliferation in vitro. (A) Effect of dh404 (200 nmol/L) on NE (20 µmol/L)-induced [^3^H]leucine uptake (left pannel) and H_2_O_2_ (100 µmol/L)-induced cell death of the cardiomyocytes infected with adenovirus of control (cont) shRNA and Nrf2 shRNA. *p<0.05 vs cont shRNA (-), n = 4. (B) Left pannel - Effect of dh404 (200 nmol/L) on Ang II (0.1 µmol/L)-, NE (20 µmol/L), and PE (20 µmol/L)-induced [^3^H]thymidine uptake in the cardiac fibroblasts. *p<0.05 vs control dh404 (-), n = 6. Right pannel – Effect of dh404 (200 nmol/L) on NE (20 µmol/L)-induced [^3^H]thymidine uptake in the cardiac fibroblasts infected with adenovirus of control (cont) shRNA and Nrf2 shRNA. *p<0.05 vs control dh404 (-), n = 4.

## Results

### Dh404 Inhibits Maladaptive Cardiac Remodeling and Dysfunction in Response to Pressure Overload

To study a therapeutic potential of dh404 for the pathological cardiac remodeling and heart failure, we examined the effect of dh404 on myocardial hypertrophy (Figure 1, Table 1), fibrosis (Figure 2) and apoptosis (Figure 3), cardiac dysfunction, and mortality (Figure 1, Table 1) in mice after 4 wk of TAC, a well-established model of pressure overload-induced pathological remodeling and dysfunction in rodents [18]. In C57BL/6J mice, cardiac hypertrophy and failure was characterized by the increased heart weight to tibial length (HW/Tibia-L) ratios, thickening of the diastolic LV posterior wall (LVPWd), increased cardiomyocyte size, (Figure 1 and Table 1), and induction of ANF and BNP fetal genes (Figure 4). In addition, TAC caused myocardial fibrosis, apoptosis, upregulation of βMHC expression and downregulation of αMHC (αMHC/βMHC switch) which collectively resulted in contractile dysfunction (i.e., reduction of myofibrillar ATPase activity, cardiac myofiber shortening and velocity of shortening), overt heart failure and significant mortality (Figures 1 and 4 and Table 1). While dh404 had little effect in the sham-operated hearts, it dramatically suppressed this maladaptive cardiac remodeling and dysfunction (Figures 1, 2, 3, 4 and Table 1). These cardiac protective effects of dh404 were dose dependent (Table 1).

### Dh404 Up-regulates Nrf2 Protein Expression, Increases Nrf2 Nuclear Translocation and Activates Nrf2-driven Gene Expression in the Heart

To determine effect of dh404 on myocardial Nrf2 activity in response to pressure overload, we assessed Nrf2 expression and nuclear translocation in sham and TAC hearts treated with vehicle or dh404 after operation. Confocal analysis revealed that myocardial expression levels of cytosolic and nuclear Nrf2 proteins were down-regulated 4 weeks after TAC (Figure 5). However, dh404 up-regulated the cytosolic and nuclear Nrf2 protein expression in sham and TAC hearts (Figure 5). In addition, dh404 increased the expression of NAD(P)H:quinone oxidoreductase (NQO-1), the most established Nrf2-driven downstream gene in the heart [18], [19] (Figure S1).

### Dh404 Suppresses Oxidative Stress in Pressure-overloaded Heart

To investigate effect of dh404 on oxidative stress in the heart after pressure overload, we measured the myocardial levels of 4-hydroxynonenal (4-HNE), a marker of the oxidative index, lipid peroxidation, and 8-hydroxydeoxyguanosine (8-OHdG), a marker of DNA damage induced by oxidative stress [18], [19]. The TAC-induced increases in 4-HNE and 8-OhdG were significantly inhibited by dh404 treatment (Figure 6).

### Dh404 Inhibits Hypertrophic or Fibrotic Responses in Cardiac Cells via Activating Nrf2

To further study molecular and cellular mechanisms of the dh404-mediated cardiac protection in vivo, we examine the effect of dh404 on Nrf2 activation as well as hypertrophic growth and death of cardiac cells in vitro. In primary cultured neonatal rat cardiomyocytes, dh404 up-regulated Nrf2 protein expression, activated mRNA expression of Nrf2 downstream genes including heme oxygenase-1 (HO-1), NAD(P)H:quinone oxidoreductase (NQO-1) and thioredoxin-1 (Txn-1), which are involved in Nrf2-mediated antioxidant defense in the heart [18], [19] (Figure S1). Dh404 inhibited norepinephrine (NE)-induced hypertrophy and H_2_O_2_-induced cell death in the cardiomyocytes infected with adenovirus of control shRNA but not in the cardiomyocytes infected with adenovirus of Nrf2 shRNA (Figure 7A). Of note, knocking down of Nrf2 enhanced the basal as well as the NE- and H_2_O_2_-induced cardiomyocyte hypertrophy and death respectively as our previously reported [18], [23]. In primary cultured neonatal rat cardiac fibroblasts, dh404 inhibited not only basal but also multiple pro-fibrotic factors including angiotensin II (Ang II)-, NE- and phenylephrine (PE)-induced cellular proliferation (Figure 7B). Knocking down of Nrf2 enhanced the basal and NE-induced proliferation in cardiac fibroblasts as our previously reported [18]; however, the loss of Nrf2 function blunted the growth inhibitory effect of dh404 in these cells (Figure 7B).

## Discussion

In this study, we provide, for the first time, evidence that chronic pressure overload results in a down-regulation of myocardial Nrf2 protein expression and administration of dh404 up-regulates myocardial Nrf2 protein expression. Moreover, we demonstrate that the dh404 treatment suppresses pressure-overload induced myocardial oxidative stress, maladaptive cardiac remodeling and heart failure via Nrf2-mediated inhibition of cardiomyocyte hypertrophy and cardiac fibroblast proliferation.

We have demonstrated that, in the absence of Nrf2, pressure overload- and Ang II-induced maladaptive cardiac remodeling and dysfunction are more severe [18], [19], thereby revealing a cardiac protective role of Nrf2 in the pathogenesis of cardiac disease. Of note, Nrf2 serves as a negative feedback regulator in these pathological settings. Consistent with our observation of the down-regulated Nrf2 expression in the failing heart of human diabetes [20], we further found in mice a down-regulation of Nrf2 expression in the failing heart after 4 wks of pressure overload. Although the molecular mechanism of the down-regulation of Nrf2 is not presently known, the decrease in Nrf2 protein expression contributes to the pathological cardiac remodeling and heart failure. Since we have demonstrated that cardiomyocyte specific over-expression of Nrf2 protects against TAC-induced maladaptive remodeling and dysfunction (unpublished data), it is conceivable that pharmacological up-regulation and activation of myocardial Nrf2 would be a therapeutic strategy for cardiac disease.

Derivatives of oleanolic acid are relatively non-toxic and cytoprotective, and have emerged as attractive drug candidates for the treatment of various diseases [27]. Accordingly, we have synthesized a novel derivative of oleanolic acid, dh404, and demonstrated that it is a potent Nrf2 activator [24]. At a molecular level, dh404 interrupts the Keap1-Cul3-Rbx1 E3 ligase complex-mediated Nrf2 ubiquitination and subsequent degradation saturating the binding capacity of Keap1 to Nrf2, thereby rendering more Nrf2 to be translocated into the nuclei to activate Nrf2-driven gene transcription. Importantly, dh404 suppresses oxidative stress in cardiomyocytes, possessing a therapeutic potential for heart disease. To support this notion, herein we demonstrated that dh404 treatment suppressed the TAC-induced down-regulation of myocardial Nrf2, and myocardial oxidative stress, fibrosis, and apoptosis, as well as the overt heart failure and increased mortality. Moreover, dh404 activated Nrf2, inhibited hypertrophy and death of primarily cultured cardiomyocytes, and attenuated proliferation of primarily cultured cardiac fibroblasts. Of note, these cardiac protective effects of dh404 were largely attenuated by knocking down of Nrf2. Because we have demonstrated that Nrf2 orchestrates redox homeostasis for the maintenance of the functional integrity of cardiomyocytes and cardiac fibroblasts thereby protecting against pathologic cardiac remodeling and dysfunction, it is plausible that dh404 exerts cardioprotective effects at least partly via activating Nrf2-driven antioxidant pathway in the heart.

Interestingly, methyl 2-cyano-3,12-dioxooleana-1,9(11)dien-28-oate (CDDO-methyl ester), an analogue of dh404, has been recently shown to activate hepatic AMP-activated protein kinase (AMPK) to improve glucose metabolism and insulin sensitivity in high fat diet-induced type 2 diabetes mice [28]. It is unclear whether CDDO-methyl or dh404 activates AMPK in the heart. However, considering a critical role of AMPK in suppressing maladaptive cardiac remodeling and dysfuction [29], [30], the possibility of dh404-driving AMPK-mediated cardiac protection is worth to be investigated. Importantly, CDDO-methyl ester has been shown in a phase 2 clinical trial to be efficacious in patients with chronic kidney disease associated with type 2 diabetes, thus providing a further impetus for its use in the treatment of chronic kidney disease [31]. As they have similar pharmacological kinetics, further characterization of dh404-mediated activation of Nrf2 signaling in the heart will thus facilitate the development of novel therapeutic strategies for the treatment of cardiac diseases.

In summary, we demonstrate that dh404 activates Nrf2 signaling and inhibits pathologic cardiac remodeling and heart failure, and appears to be an attractive therapeutic candidate for the treatment of cardiac disease.

## Supporting Information

Figure S1 **Effect of dh404 on Nrf2 expression and activation in cardiomyocytes and myocardium.** Rat neonatal cardiomyocytes were isolated and cultured. (A) Upper left panel - representative images of Western blot analysis of Nrf2 expression in the cardiomyocytes. Lower panel - Q-PCR analysis of mRNA expression of Nrf2 downstream genes including HO-1, NQO-1 and Txn-1 in the cardiomyocytes. *p<0.05 vs control (-), n = 6. Male C57BL/6J mice (n = 5) at age of 14 weeks were administrated with or without single gavages of dh404 (10 mg/kg) or vehicle (sesame oil). Hearts were harvested at 0, 6 h, 24 h, and 48 h after the treatment. RNAs from left ventricles of these hearts were subjected to Q-PCR analysis of the expression of NAD(P)H:quinone oxidoreductase (NQO-1), the most established Nrf2-driven downstream gene in the heart as our previously described [18], [19].
(TIFF)
