# Correction: Triterpenoid Dihydro-CDDO-Trifluoroethyl Amide Protects against Maladaptive Cardiac Remodeling and Dysfunction in Mice: A Critical Role of Nrf2

**DOI:** 10.1371/journal.pone.0314793

**Published:** 2024-11-27

**Authors:** Yifan Xing, Ting Niu, Wenjuan Wang, Jinqing Li, Siying Li, Joseph S. Janicki, Stacey Ruiz, Colin J. Meyer, Xing Li Wang, Dongqi Tang, Yuxia Zhao, Taixing Cui

In Table 1, the Echochardiographic values of LVIDs should not have been a duplicate of what is in LVIDd to both Sham and TAC groups. Please see the correct Table 1 here. Please see the correct Table 1 here.

**Table 1:** Echocardiography and pathology of mice treated with and without dh404 for 4 wks after TAC.

		Dh404 (mg/kg/d)			
		0	5	10	20
**Sham**	**Echocardiography** (n)	10	4	7	4
	IVSd (mm)	0.77 ± 0.04	0.74 ± 0.05	0.84 ± 0.05	0.83 ± 0.03
	LVIDd (mm)	4.06 ± 0. 13	4.14 ± 0.13	3.96 ± 0.22	3.93 ± 0.23
	LVIDs (mm)	3.18 ± 0.15	3.21 ± 0.21	3.04 ± 0.24	3.17 ± 0.21
	LVPWd (mm)	0.69 ± 0.03	0.64 ± 0.02	0.68 ± 0.02	0.65 ± 0.05
	FS (%)	21.81 ± 1.78	22.53 ± 3.20	24.03 ± 2.01	19.35 ± 2.00
	**Pathology** (n)	8	4	6	3
	BW (g)	25.43 ± 0.79	26.05 ± 1.46	25.53 ± 1.10	25.03 ± 0.50
	HW/Tibia-L (g/cm)	7.52 ± 0.29	7.61 ± 0.53	7.40 ± 0.34	7.02 ± 0.17
	LW/Tibia-L (g/cm)	10.61 ± 0.29	10.21 ± 0.84	11.03 ± 0.23	8.96 ± 0.82
**TAC**	**Echocardiography** (n)	16	5	13	8
	IVSd (mm)	1.14 ± 0.04^a^	0.91 ± 0.06^a^^,^^b^	0.99 ± 0.03^a^^,^^b^	0.94 ± 0.05^a^^,^^b^
	LVIDd (mm)	5.11 ± 0.14^a^	4.73 ± 0.17^a^^,^^b^	4.73 ± 0.11^a^^,^^b^	4.71 ± 0.17^a^^,^^b^
	LVIDs (mm)	4.76 ± 0.16^a^	3.36 ± 0.22^a^^,^^b^	4.25 ± 0.12^a^^,^^b^	4.01 ± 0.18^a^^,^^b^
	LVPWd (mm)	1.08 ± 0.04^a^	0.92 ± 0.06^a^^,^^b^	0.90 ± 0.03^a^^,^^b^	0.90 ± 0.08^a^^,^^b^
	FS (%)	7.02 ± 1.10^a^	7.88 ± 1.40^a^^,^^b^	10.42 ± 0.94^a^^,^^b^^,^^c^	14.77 ± 1.97^a^^,^^b^^,^^c^^,^^d^
	**Pathology** (n)	16	5	13	8
	BW (g)	22.85 ± 0.73^a^	23.56 ± 0.80^a^	22.34 ± 0.67^a^	20.65 ± 0.97^a^
	HW/Tibia-L (g/cm)	15.54 ± 0.48^a^	12.23 ± 0.65^a^^,^^b^	11.68 ± 0.42^a^^,^^b^	11.56 ± 0.37^a^^,^^b^
	LW/Tibia-L (g/cm)	26.55 ± 2.23^a^	15.79 ± 4.30^a^^,^^b^	17.23 ± 1.21^a^^,^^b^	21.17 ± 2.76^a^^,^^b^

Two dimensional echocardiography-guided M-mode echocardiography and pathology were performed 4 weeks after the initiation of TAC or sham surgery in C57BL/6J mice treated with vehicle or dh404 as indicated.

Abbreviations: IVSd, interventricular septal thickness at end-diastole; LVIDd, left ventricular internal dimension at end-diastole; LVIDs, left ventricular internal dimension at end-diastole; LVPWd, left ventricular posterior wall thickness at end-diastole; FS, fractional shortening; BW, body weight; HW/Tibia L, heart weight/tibia length ratio; LW/Tibia-L, lung weight/tibia length ratio.

^a^p<0.05 vs sham (0);

^b^p<0.05 vs TAC (0);

^c^p<0.05 vs TAC + dh404 (5 mg/kg/d);

^d^p<0.05 vs TAC + dh404 (10 mg/kg/d)
