# Supplementary figures and images for: Triterpenoid Dihydro-CDDO-Trifluoroethyl Amide Protects against Maladaptive Cardiac Remodeling and Dysfunction in Mice: A Critical Role of Nrf2
Source: PLoS One. 2012 Sep 17;7(9):e44899. doi: 10.1371/journal.pone.0044899 (PMC3444497; doi:10.1371/journal.pone.0044899)

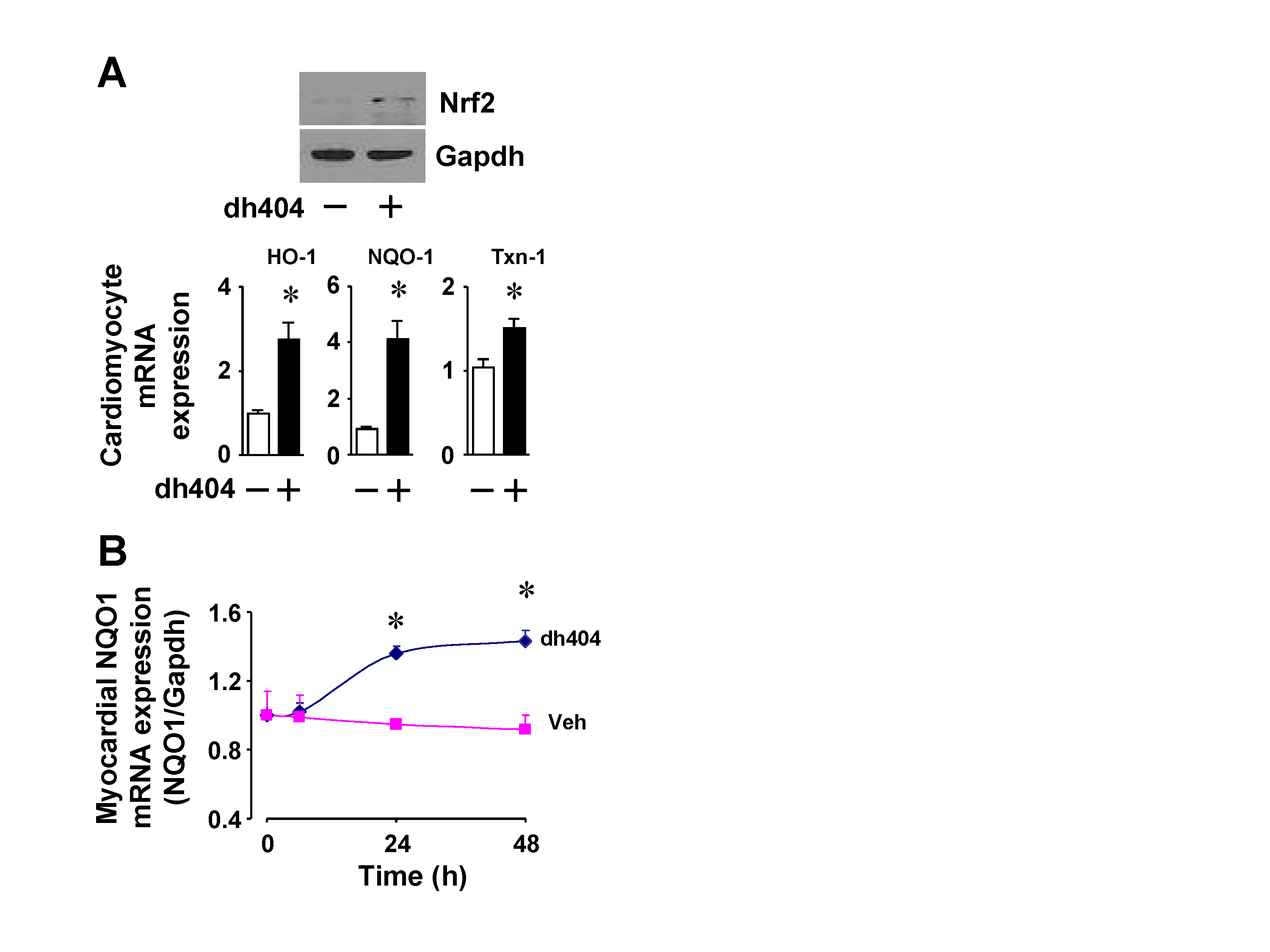

Supplement: Figure S1 — Effect of dh404 on Nrf2 expression and activation in cardiomyocytes and myocardium. Rat neonatal cardiomyocytes were isolated and cultured. (A) Upper left panel - representative images of Western blot analysis of Nrf2 expression in the cardiomyocytes. Lower panel - Q-PCR analysis of mRNA expression of Nrf2 downstream genes including HO-1, NQO-1 and Txn-1 in the cardiomyocytes. *p<0.05 vs control (-), n = 6. Male C57BL/6J mice (n = 5) at age of 14 weeks were administrated with or without single gavages of dh404 (10 mg/kg) or vehicle (sesame oil). Hearts were harvested at 0, 6 h, 24 h, and 48 h after the treatment. RNAs from left ventricles of these hearts were subjected to Q-PCR analysis of the expression of NAD(P)H:quinone oxidoreductase (NQO-1), the most established Nrf2-driven downstream gene in the heart as our previously described [18], [19]. (TIFF) [file pone.0044899.s001.tiff]
